# Trends in the Rates of Peripartum Hysterectomy and Uterine Artery Embolization

**DOI:** 10.1371/journal.pone.0060512

**Published:** 2013-04-02

**Authors:** Geum Joon Cho, Log Young Kim, Hye-Ri Hong, Chang Eun Lee, Soon-Cheol Hong, Min-Jeong Oh, Hai-Joong Kim

**Affiliations:** 1 Department of Obstetrics and Gynecology, Korea University Guro Hospital, Korea University College of Medicine, Seoul, Korea; 2 The Health Insurance Review and Assessment Service of Korea, Seoul, Korea; 3 Dongnam Health University, Suwon, Korea; Baylor College of Medicine, United States of America

## Abstract

The objective of this study was to determine the trends in national rates of peripartum hysterectomy (PH) and uterine arterial embolization (UAE) in Korea. We used data collected by the Health Insurance Review & Assessment Service of Korea and analyzed data from patients who gave birth during the period from 2005 to 2008. There were 1785,178 deliveries during the study period, including 2636 cases of PH (1.48 per 1000 deliveries). The PH rate in 2005 was 1.57 per 1000 deliveries and in 2008 it was 1.33 per 1000 deliveries. UAE was performed in 161 women (incidence, 0.38 per 1000 deliveries) and 447 women (incidence, 0.98 per 1000 deliveries) in 2005 and 2008, respectively. In Korea, the rate of PH decreased slightly, while the rate of UAE rate increased dramatically during the period from 2005 to 2008. Further studies are needed to evaluate the effects of UAE on the rate of PH performed.

## Introduction

A peripartum hysterectomy (PH) is a procedure performed at the time of delivery or in the immediate postpartum period as a life-saving measure in response to severe postpartum hemorrhage that does not respond to any other interventions [1]. However, PH is one of the most severe complications in obstetrics and results in significant maternal mortality and morbidity [2], [3]. Moreover, PH results in a permanent loss of future childbearing opportunities [4]. Its reported rate varies from 0.24 per 1000 deliveries [5] to 5.09 per 1000 deliveries [6]. Moreover, the results from studies that evaluated trends in the number of peripartum hysterectomies performed over time are mixed [4], [7]–[10]. These discrepancies between studies may be due to the fact that these studies were conducted in single institutions and the sample sizes were therefore small. Furthermore, these data are not appropriate to obtain reliable nationwide rate estimates [4]. In fact, only a handful of studies have examined PH rates in total populations of a nation or region [1], [4], [10], [11].

An alternative to PH to manage postpartum hemorrhage is selective uterine artery embolization (UAE). UAE is a reliable, safe, and minimally invasive procedure that has consistently been demonstrated to have success rates of over 90% with regard to achieving hemostasis [12], [13]. One of the major advantages of UAE in the treatment of postpartum hemorrhage is its potential to avoid hysterectomy and thereby preserve a woman's future childbearing options [14]. However, few studies have evaluated trends in the UAE rate and its association with the PH rate.

Our aims in this study were to describe nationwide trends in PH and UAE in Korea and identify the risk factors of PH.

## Materials and Methods

Data were collected from the Korea National Health Insurance Claims Database of the Health Insurance Review & Assessment Service (HIRA) for the period 2005 to 2008. Under the National Health Insurance System, Koreans are entitled to medical coverage as either an employee or a member of a community. Healthcare providers are required under the health insurance policies of HIRA to provide a review of the medical costs incurred. Accordingly, the HIRA database contains information on claims for approximately 50 million Koreans [15]. The study protocol was approved by the institutional review boards of the Health Insurance Review & Assessment Service (IRB No. HIRA-1587)(10/November/2010). All data were de-identified by HIRA. The diagnosis and procedure codes from the International Classification of Diseases, 10th revision, were used to identify all women who gave birth during the study period and women who underwent PH or UAE. Cases of PH included women who underwent a vaginal or cesarean delivery in combination with an abdominal hysterectomy (either a total or subtotal abdominal hysterectomy). Cases of UAE were defined as UAE and delivery occurring during the same hospitalization event. Women with a concomitant diagnosis of a malignancy were excluded from the analysis.

To identify the risk factors for PH and UAE, demographic characteristics, namely age, multiple pregnancy (defined as twins or higher-order gestations), parity (primiparous or multiparous), placenta previa, and obstetric procedures used for delivery (cesarean delivery, instrumental delivery, induction of labor) were obtained.

The rates of PH and UAE were calculated per 1000 deliveries. The rates of several factors related to PH were calculated per 1000 deliveries. The rates of PH and UAE related to each factor were calculated per 1000 deliveries. Data from each year were evaluated individually and then compared to identify emerging trends. Trends over time were assessed by entering year as a single term with equally spaced category scores into logistic regression models.

Multivariate logistic regression analysis was carried out with PH and UAE as the final outcome. Odds ratios (ORs) and 95% confidence intervals (CIs) were calculated using logistic regression. A *P*-value of less than 0.05 was considered statistically significant. Statistical analyses were performed using SPSS software, version 12.0 (SPSS Inc., Chicago, IL, USA).

## Results

For the study period of 2005–2008, a total of 1785,178 deliveries were recorded and a total of 2636 PH were performed in Korea, corresponding to a rate of 1.48 per 1000 deliveries. A total of 1239 UAE were performed, translating to a rate of 0.69 per 1000 deliveries. Table 1 shows trends in PH and UAE rates during the study period. The PH rate decreased slightly but significantly from 1.57 per 1000 deliveries in 2005 to 1.33 per 1000 deliveries in 2008 (*P*<0.001). There was a noticeable and significant increase in the UAE rate from 0.38 per 1000 deliveries in 2005 to 0.98 per 1000 deliveries in 2008 (*P*<0.001).

**Table 1:** The numbers and rates^a^ of PHs and UAEs and the *P*-values for these rates during the study period.

	PH		UAE	
Year	Number	Rate	Number	Rate
2005	664	1.57	161	0.38
2006	660	1.51	258	0.59
2007	706	1.50	373	0.79
2008	606	1.33	447	0.98
Overall	2636	1.48	1239	0.69
*P*-value for trend		<0.001		<0.001

PH, peripartum hysterectomy; UAE, uterine artery embolization.

The *P*-value was evaluated by logistic regression analysis.

a Rate was calculated per 1000 deliveries.

The case numbers and rates of risk factors for PH and UAE and their trends are shown in Table 2. The rates of cesarean delivery, multiparity, and instrumental delivery decreased during the study period. However, the rates of multiple pregnancy, induction of labor, and placenta previa increased. Table 3 shows case numbers and rates of PH for each risk factor and their trends during the study period. The rate of PH for instrumental delivery increased, but the rate of PH for other factors did not change significantly during the study period. The rate of UAE for each risk factor increased during study period although this was not statistically significant (Table 4).

**Table 2:** The numbers and rates^a^ of risk factors for PH and UAE and their trends (*P*-values) during the study period.

	2005		2006		2007		2008		Overall		
	Number	Rate	Number	Rate	Number	Rate	Number	Rate	Number	Rate	*P*-value for trend
Cesarean delivery	157,125	372.02	157,397	361.03	171,296	363.70	164,802	361.51	650,620	364.46	<0.001
Multiparity	204,634	484.51	209,625	480.83	219,705	466.48	205,858	451.57	839,822	470.44	<0.001
Multiple pregnancy	5265	12.47	5238	12.01	6909	14.67	6763	14.84	24,175	13.54	<0.001
Induction of labor	80,098	189.65	87,637	201.02	96,704	205.32	93,392	204.87	357,828	200.44	<0.001
Instrumental delivery	28,803	68.20	27,807	63.78	28,775	61.09	27,181	59.62	112,566	63.06	<0.001
Placenta previa	3121	7.39	3424	7.85	3940	8.37	3893	8.54	14,378	8.05	<0.001

PH, peripartum hysterectomy; UAE, uterine artery embolization.

The *P*-value was evaluated by logistic regression analysis.

a Rates were calculated per 1,000 deliveries.

**Table 3:** The numbers and rates^a^ of PH according to each risk factor and their trends during the study period.

	2005		2006		2007		2008		Overall		
	Number	Rate	Number	Rate	Number	Rate	Number	Rate	Number	Rate	*P*-value for trend
Cesarean delivery	463	2.95	418	2.66	498	2.88	428	2.60	1,807	2.78	0.202
Multiparity	408	1.99	378	1.80	417	1.90	355	1.72	1,558	1.86	0.179
Multiple pregnancy	30	5.70	45	8.59	69	9.99	41	6.06	185	7.65	0.109
Induction of labor	79	0.99	81	0.92	92	0.95	68	0.74	320	0.89	0.424
Instrumental delivery	18	0.62	17	0.61	29	1.01	33	1.21	97	0.86	0.002
Placenta previa	140	44.86	130	37.97	154	39.09	135	34.68	559	38.88	0.574

PH, peripartum hysterectomy.

*p*-values were evaluated by multivariate logistic regression analysis.

a Rates were calculated per 1,000 deliveries with each risk factor.

**Table 4:** The numbers and rates^a^ of UAE according to each risk factor and their trends during the study period.

	2005		2006		2007		2008		Overall		
	Number	Rate	Number	Rate	Number	Rate	Number	Rate	Number	Rate	*P*-value for trend
Cesarean delivery	88	0.56	123	0.78	169	0.99	225	1.37	605	0.93	0.820
Multiparity	65	0.32	114	0.54	163	0.74	178	0.86	520	0.62	0.540
Multiple pregnancy	11	2.09	19	3.63	26	3.76	43	6.36	99	4.10	0.188
Induction of labor	28	0.97	43	1.55	83	2.88	86	3.16	240	2.13	0.639
Instrumental delivery	11	0.14	21	0.24	24	0.25	27	0.29	83	0.23	0.433
Placenta previa	27	8.65	39	11.39	61	15.48	63	16.18	190	13.21	0.672

UAE, uterine artery embolization.

*p*-values were evaluated by multivariate logistic regression analysis.

a Rates were calculated per 1,000 deliveries with each risk factor.

Multivariate-adjusted ORs for PH are shown in Table 5. Age, multiparity, multiple pregnancy, cesarean delivery, induction of labor, instrumental delivery, and placental previa were associated with an increased risk of PH. The highest risk was noted in women with placenta previa (OR 22.710, 95% CI 20.547%–25.100%). Mutlitvariate-adjusted ORs for UAE are shown in Table 6. Age, multiple pregnancy, cesarean delivery, induction of labor, instrumental delivery, and placental previa were associated with an increased risk of UAE, but multiparity was associated with a decreased risk of UAE.

**Table 5:** Adjusted ORs for the risk of PH.

	OR^a^	95% CI
Age	1.038	1.035–1.041
Cesarean delivery	3.346	2.974–3.765
Multiparity	1.075	1.573–1.849
Multiple pregnancy	4.232	3.623–4.945
Induction of labor	1.721	1.480–2.002
Instrumental delivery	1.929	1.538–2.418
Placenta previa	22.710	20.547–25.100

OR, odds ratio; PH, peripartum hysterectomy.

a ORs were adjusted for all variables in the table.

**Table 6:** Adjusted ORs for the risk of UAE.

	OR^a^	95% CI
Age	1.031	1.025–1.037
Cesarean delivery	1.410	1.140–1.743
Multiparity	0.753	0.639–0.888
Multiple pregnancy	5.886	4.388–7.896
Induction of labor	1.492	1.167–1.909
Instrumental delivery	1.450	1.011–2.079
Placenta previa	18.959	14.932–24.072

OR, odds ratio; UAE, uterine artery embolization.

a ORs were adjusted for all variables in the table.

## Discussion

To the best of our knowledge, this study is the first to report nationwide trends in PH and UAE rates. The PH rate decreased substantially from 2005 to 2008 in Korea. Although the reason for this downward trend is unclear, there are several possible explanations. Multiple factors are likely to affect the trend in the PH rate, and the observed trend may reflect complex changes in the rates of risk factors. In this study, significant risk factors for PH were age, cesarean delivery, multiparity, multiple pregnancy, induction of delivery, instrumental delivery, and placenta previa, consistent with the results from previous studies [4], [10], [16]–[18]. However, trends in the rates of individual risk factors varied during the study period. The rate of multiparity decreased and the rate of cesarean delivery, which is now cited by the majority of modern reviews as the major risk factor for PH [4], [11], [17], [19], and which was the most common factor for PH among various risk factor in this study, also decreased in contrast to the trend reported by other studies [20], [21]. However, in cases of abnormal placentation such as placenta previa, which was a significant factor for PH with the highest OR in this study, consistent with other studies [1], [2], [7], [9], its rate increased without significant changes in the PH rate. Rates of other risk factors showed increases or decreases in our study, but their effects on trends in the PH rate were likely minimal because of the small number of cases. Therefore, the downward trend in PH rate observed in our study might reflect changes in the rates of the risk factors described above, especially the decreased rate of cesarean delivery. Furthermore, increased use of UAE may also lead to a decrease in the PH rate, as UAE has been demonstrated to have a success rate of over 90% for the treatment of postpartum hemorrhage [12], [13]. We hypothesized that given the same indications; UAE would be preferred over PH, resulting in a decrease in the PH rate and an increase in the UAE rate over time. In particular, the PH rate for each risk factor should decrease over time. It is interesting to note that even though the rate of PH decreased slightly, the PH rate for each risk factor did not change significantly despite the significant and dramatic increase in the UAE rate. These results indicate that the decrease in PH rate demonstrated may be due to a decreased rate of significant risk factors for PH including cesarean delivery, rather than increased use of UAE. The immediate availability of UAE remains a challenge, especially in community hospitals in rural areas or smaller community hospitals, because UAE procedures are performed by specially trained interventional radiologists and require a well-equipped radiology suite [14]. Otherwise, in this study, the If the resources are available, it is likely that UAE will increasingly be used with a low threshold for a prompt aggressive response. It may partially explain the results that the rate of UAE for each risk factor increased although this was not statistically significant and the UAE rate increased sharply but the PH rate decreased slightly. Moreover, UAE is usually performed when the mother is hemodynamically stable [22], [23]. For example, if there is a massive hemorrhage during caesarean section the patient would be considered hemodynamically unstable and would not be suitable for UAE since UAE procedures require some time for preparation; therefore, in some obstetric situations with the potential for massive hemorrhage, including placenta percreta, planned PH is the preferred delivery strategy [24], [25]. Therefore, although the UAE rate increased rapidly during study period, its effects on the decrease in the PH rate were minimal. However, decreases in the PH rate caused by other factors not examined in this study may have contributed to the observed PH rate. Further studies are required to evaluate the exact effects of UAE on PH rate.

Studies of trends have provided a mixed picture with increase (8,10), decrease (7,9), or no change (4).In our study, the trend in the PH rate was evaluated only for the most recent 4 years. Moreover, our data collection methods were different to those used in previous studies. Therefore, direct comparison of our results with those of previous studies is not possible.

In our study, the overall PH rate was 1.48 per 1000 deliveries, comparable with some studies [2], [26] but higher than others [1], [4], [5], [8], [9], [11], [19]. This may be due to the high cesarean section rate (36.45%) demonstrated in this study, as the rate of cesarean sections is tightly linked to the PH rate [4], [11], [17], [19]. The high PH rate in our study may also be due to our study design, such as our definition of the time period for PH [10], [11].

Several limitations should be kept in mind when interpreting our findings. First, this study was based on insurance claim data in the Korea National Health Insurance Claims Database, which is a database that was designed for cost claim issues, not research. Thus, the main limitation remains the validity of the data in this database. However, KNHI data has been validated in a previous study [27]. Another limitation of our study is that we were not able to identify the standard hemodynamic parameters or the indications for PH and UAE from each hospital, and the specific individual characteristics of maternities, because the data is based on insurance claims and we did not perform comprehensive chart reviews.

Limitations of this study also include the short study period (years 2005–2008), the data collected from the Korea National Health Insurance Claims Database of the Health Insurance Review & Assessment Service (HIRA) are strictly regulated since 2005 in terms of accuracy and reliability hence we decided to use data from 2005. Secondly, since the data were obtained upon claims, at least one year was required to receive the data for year 2008. Therefore, at the time we designed our study, data up to year 2008 was the most recent available and accurate data.

Nevertheless, we included all deliveries and PH procedures performed in Korea in our study. Therefore, our results are unlikely to have been influenced by the type of hospital or the characteristics of the individual patients and physicians. The multi-center nature of our study can explain the discrepancy between our study and another study that reported a high rate of PH in Korea (3.25 per 1000 deliveries for Korean women) [17], as this latter study was performed in a single referral hospital, which is more likely to handle a greater proportion of complicated deliveries [4].

In conclusion, during the period from 2005–2008 in Korea, the PH rate decreased slightly, but the UAE rate increased sharply. Further studies are needed to evaluate the long-term trends in the PH rate and the effects of UAE on the PH rate.
